# Two step porosification of biomimetic thin-film hydroxyapatite/alpha-tri calcium phosphate coatings by pulsed electron beam irradiation

**DOI:** 10.1038/s41598-018-32612-x

**Published:** 2018-09-28

**Authors:** Bryan W. Stuart, James W. Murray, David M. Grant

**Affiliations:** Advanced Materials Research Group, Faculty of Engineering, University of Nottingham, Nottingham, UK

## Abstract

Here we show a new and effective methodology for rapid/controllable porosification of thin-film ceramics, which may be applied in medical devices/electronics and membrane nano-filtration. Dense hydroxyapatite applied to Ti6Al4V by plasma-assisted PVD was electron-beam irradiated to induce flash melting/boiling. Deposited coatings contained amorphous and nano-crystalline/stoichiometric hydroxyapatite (~35 nm). Irradiation (voltages 13–29 kV) led to ablation (up to 45% mass loss) and average/maximum pore areas from (0.07–1.66)/(0.69–92.53) μm^2^, mimicking the human cortical bone. Vitrification above 1150 °C formed (~62–30 nm) crystallites of α-Tri Calcium Phosphate. Unique porosification resulted from irradiation-induced sub-surface boiling and limited thermal conductivity of hydroxyapatite, causing material to expand/explode through the more quickly solidified top surface. Commercially applicable, roughened Ti6Al4V exacerbated the heating and boiling explosion phenomenon in certain regions, producing an array of pore sizes. Scaffold-like morphologies were generated by interconnection of micron/sub-micron porosity, showing great potential for facile generation of a biomimetic surface treatment for osseointegration.

## Introduction

Physical Vapour Deposition (PVD) is suitable for the production of controllable coating layers due to its versatility in producing thin (sub-micron to micron level) amorphous or crystalline films with highly adherent and customisable compositions and microstructures. Here, a new two-step methodology has been developed using low-energy (10–40 keV) high-current (10–25 kA)^1^ pulsed electron beam irradiation (LHEB) to porosify hydroxyapatite (HA) thin film coatings. LHEB has been used for materials processing applications including surface alloying, micro-polishing, surface annealing and controlling crystallinity. Irradiation causes melting of the first few microns depth of material and has typically been used to reduce roughness via melting of asperities however crater formation (typically sparsely distributed) is sometimes an unwanted side effect of the technique, thought to be caused by sub-surface melt pools within the depth of the section. The melt pools have been theorised to nucleate at micro-irregularities including grain/phase boundaries or precipitates in the material, resulting in eruption through the surface, leaving a crater morphology behind^2^. Given the ability of the LHEB process to irradiate a large surface area (60 mm diameter), maintain its energy density at high angles to treat curved surfaces^3,4^, as well as take place within 2–4 µs^1^, the technique was investigated here as a novel method of porosification in HA coatings which may be more widely applicable to ceramics in gas/liquid membrane applications or electronics.

Osteogenic coatings facilitate protein attachment and stimulate osteoblast cell differentiation to accelerate secretion of collagen fibrils and mineralisation of an extracellular matrix^5–8^. Commercial thermal spray methodologies such as plasma sprayed Hydroxyapatite have been used since the 1980′s for osteoconductive orthopaedic coatings for implant osseointegration. The motivation was to mimic the mineral phase of the human cortical bone by maintaining a stoichiometric HA structure with a Ca/P ratio of 1.67 formulated as (Ca_10_(PO_4_)_6_(OH)_2_)^9–11^. However, the Ca/P particles injected through the thermal spraying flame result in splats that can exceed 2000 °C^12,13^. When combined with large cooling gradients and thermal expansion this leads to poor adhesion, undesirable porosity and variations in crystallinity/phase composition^12^. Limitations such as inability to tailor crystallite size to mimic the natural bone by plasma spraying means that commercial HA remains poorly biomimetic, requiring a significant step change towards precision manufacturing and nano scale customisation. It can be noted that natural cortical bone has an average porosity of <5% and HA crystallite size of 20 nm, lending to resorption within the body to enable cyclical bone remodelling^6,7^.

In contrast, HA coatings have been deposited by PVD methods such as plasma assisted sputtering since 1992^14,15^. Sputtering has been extensively implemented for HA and bioactive glasses owing to favourable adhesion as compared to all other methods^16–22^ and has extended to co-deposited ion-doped HA layers, containing osteogenic and antimicrobials ions such as Silicon, Strontium, Titanium, Silver, Gallium^23,24^ as therapeutic ion leaching phosphate/bioactive silicate based coating layers^25,26^.

Implant topography and porosity has shown equal importance for cellular habitation and surface attachment^27,28^. A clinical study assessed the pore size distribution of healthy human cortical bone of the femur and found that 41% of the pores existed in the 7.5–50 nm range, with 31% in the 1–15 micron range^29^. Hydroxyapatite coatings with nano-micro porosity have been fabricated by self-assembly of core shell nanoparticles during which nano porosity facilitated filopodia attachment attributed to enhanced osseointegrative capabilities^30^.

The work presented here shows the results of the approach to develop a two-step process of Magnetron Sputtering/Electron Beam Irradiation to demonstrate a rapidly producible, controllable interconnected porosity in thin-films of HA.

## Results and Discussion

### Electron Beam Induced Phase and Compositional Modifications

**Figure 1:**
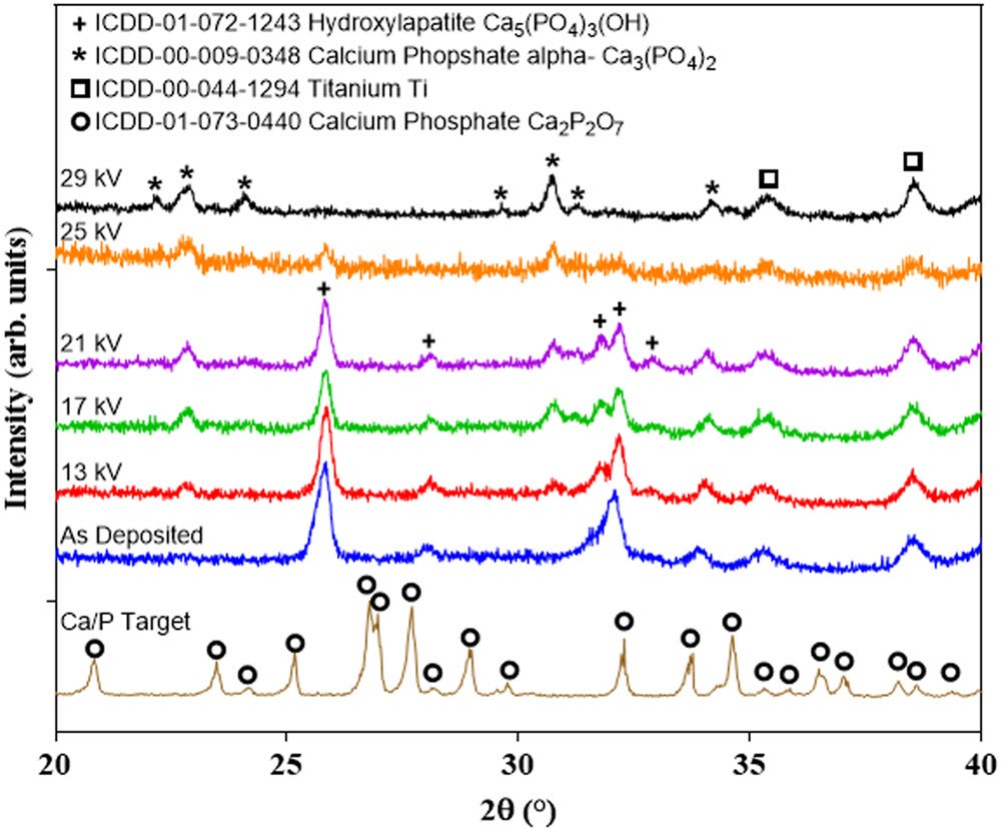
XRD spectra of the target, AD and electron irradiated samples. Coatings were deposited as crystalline HA with preferred orientation of the (002) plane. Electron irradiation formed a α-TCP phase, which became dominant following irradiation of 29 kV.

In this two step process there is novelty not only in the combination but the preparation of the first deposited layer. Here we have developed alternative targets based on Ca/P glasses combined with appropriate sputtering conditions to produce crystalline coatings (Fig. 1). By comparison, due to the near instantaneous nature of atomic condensation, sputtering has often been reported to produce fully amorphous Ca/P structures, requiring a post deposition heat treatment in excess of 650 °C to tailor crystallinity towards biomimetic and physiologically stable layers^20,31–35^. The disadvantage of post heating is a tendency to propagate cracks and cause interfacial detachment from differential thermal expansion between the substrate and coating^25^. Lopez *et al*. similarly showed single step production of crystalline HA by right angle sputtering whereby two parallel magnetrons enhanced plasma energy at the combination edge of the plasma sheath, increasing electron density to nucleate a crystalline phase during condensation^36^. Here, by using a target based on Ca_2_P_2_O_7_ crystals (Fig. 1) combined with the stated deposition conditions, crystalline HA coatings were deposited without any post treatment in readiness for secondary rapid porosification. Specifically the as deposited (AD) layer was found to contain crystalline HA with c-axis orientation indicated by preferred alignment in the direction of the (002) plane located at 26° (2θ)^37^. A 0.1° (2θ) shift of bragg peaks from the ICDD specification was observed in the HA phase.

The second step novelty involved using LHEB to nucleate/grow crystalline biomimetic Ca:P phases as an alternative to an otherwise lengthy post heat treatment previously used by Van der wal *et al*.^35^. All substrates were medical grade Ti6Al4V, surface roughened via alumina grit blasting, prepared as in reference^25^. The emerging peaks following electron irradiation of 13, 17 and 21 kV at 23 and 31° (2θ) were associated with formation of a unique and exciting presence of bioactive α-Tri Calcium Phosphate phase, which prevailed at the expense of HA following 29 kV irradiation (Fig. 1). Crystallite sizes were approximated using the Scherrer equation for HA and α-TCP (Fig. 1 and Table 1) by analysis of Bragg diffraction peaks at 26 and 23° (2θ) respectively showing the formation of α-TCP following 13 kV irradiation and elimination of the HA phase following 29 kV. Increase in voltage led to an increase in HA size from 35 to 80 nm and simultaneous reduction in crystallite size ranging from 62 to 30 nm for α-TCP.

**Table 1:** Crystal sizes were calculated for HA and α-TCP using the Scherrer equation by measuring the Full Width at Half Maximum (FWHM) for peaks positioned at ~25.8° and 22.8° (2θ).

Hydroxyapatite						
Sample	AD	13 kV	17 kV	21 kV	25 kV	29 kV
Position (°) (2θ)	25.8	25.84	25.82	25.82	25.83	—
FWHM	0.36	0.29	0.23	0.26	0.26	—
Crystal size (nm)	35.3	50.6	80.7	62.2	62.2	—
**α–tri-calcium phosphate**						
**Sample**	**AD**	**13 kV**	**17 kV**	**21 kV**	**25 kV**	**29 kV**
Position (°) (2θ)	—	22.82	22.85	22.84	22.8	22.82
FWHM	—	0.26	0.28	0.28	0.4	0.34
Crystal size (nm)	—	61.9	53.7	53.7	29.9	38.4

Single shot irradiation between 13–25 kV altered the coating phase composition from HA to a mixture of HA and α-TCP, observed by XRD (Fig. 1). At 29 kV, HA appeared to be entirely converted to α-TCP. The electron pulse was emitted over an estimated timespan of several µs, resulting in near-instantaneous re-melting and quenching of the surface. To investigate phase transformation Kreidler and Hummel *et al*. constructed a phase diagram of CaO-P_2_O_5_ composites which explains the phase transformation observed here from HA to α-TCP. An α-TCP + Liquid phase may be precipitated in CaO-P_2_O_5_ (62.5:37.5 mol%) structure when heated to above 1125 °C if CaO exceeds 55 mol% within the CaO-P_2_O_5_ structure^38^. To avoid transition to *β-*TCP and maintain an α-TCP + amorphous structure, rapid cooling was required and naturally occurred due to localised melting and rapid cooling of the thin layer dissipating heat to remaining unaffected coating beneath the modified layer, the Ti6Al4V substrate acting as a heat sink, as well as radiating heat from the surface. This phase transformation to the metastable phase in a coating was extraordinary, and may only be obtained by flash melting induced by high energy irradiation^38^. The α-TCP phase is considered a bioactive Ca:P derivative and has been previously produced by melt-quenching, used in bone cements^39^, however it has a unique presence here within orthopaedic coating compositions. The inherent bioactivity is due to the metastable nature of α-TCP and therefore its susceptibility to convert to HA by hydrolysis in physiological media^39^. Conventional thermal spray struggles to maintain the α-phase due to slow bulk cooling rates as compared to PVD.

**Figure 2:**
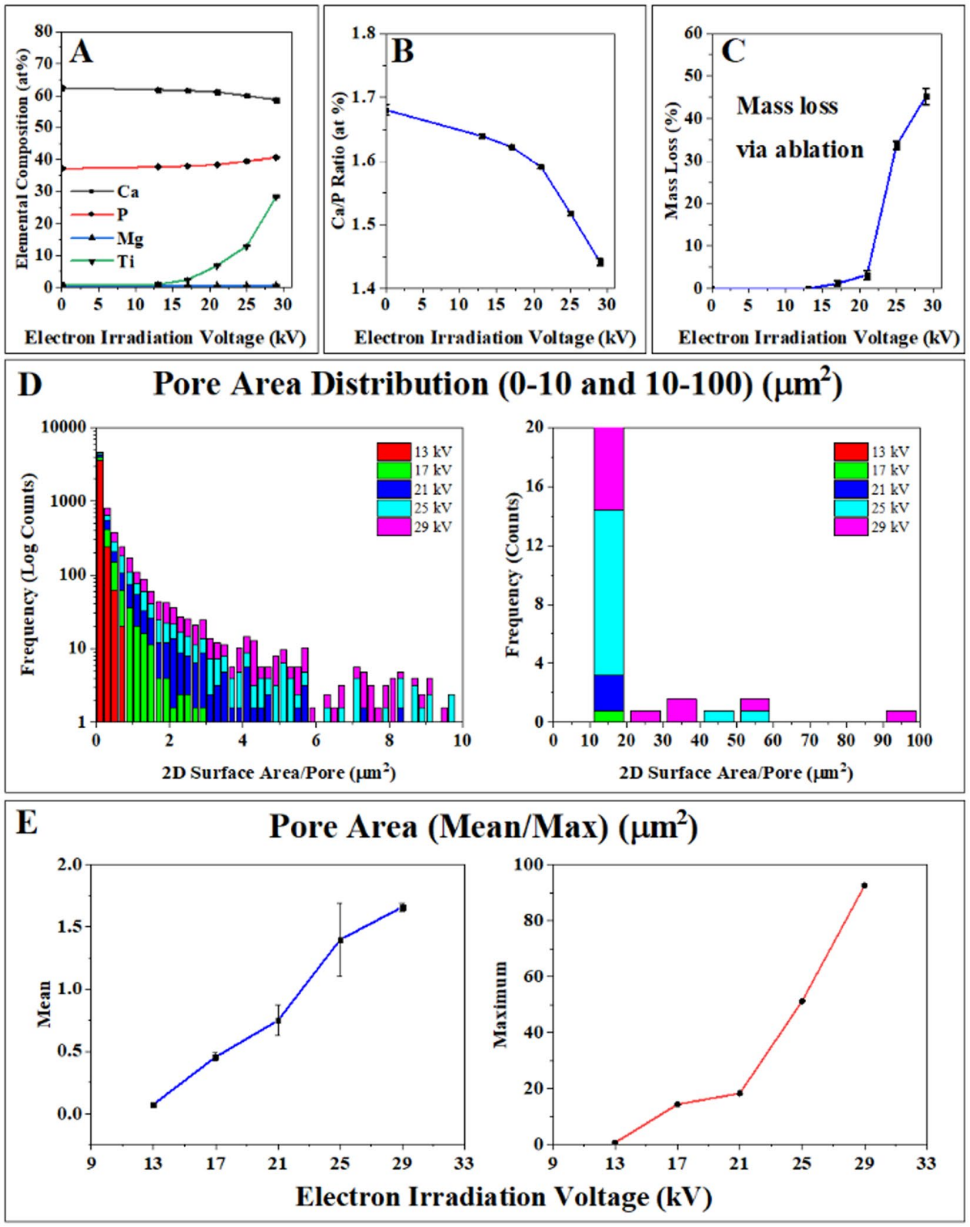
(**A**) Elemental compositions by EDX (**B**) Ca:P Ratios of AD and post electron irradiated samples. An increase in Ca:P ratio from the stoichiometries of HA (1.67) towards TCP (1.5) was observed whilst increasing Titanium signal was attributed to coating porosity. (**C**) Tracking of the sample weights showed mass losses associated with electron irradiation as an indication of coating porosity following ablation. (**D**) All pore surface areas were measured over a total of 1200 μm^2^ from 5 randomly selected regions. (E) Average and maximum pore areas were shown, relative to irradiation voltage.

The elemental composition of the target, AD and irradiated samples were assessed by EDX (Fig. 2B). A Ca_2_P_2_O_7_ quenched ceramic powder with a Ca:P ratio of ~1 was sputtered to produce an as deposited surface film with an increased Ca:P ratio of ~1.67, consistent with stoichiometric HA (Fig. 2C) as stated above. Following irradiation at 13, 17, 21, 25 and 29 kV the Ca:P ratios of the coating layers gradually diminished towards ~1.43, consistent with Ca deficient Tri Calcium Phosphate (TCP) which coincided with XRD results presented to show formation of an α-TCP phase (Fig. 1).

The EDX results showed an increase in titanium from 1.0 to 30 at%, consistent with the increase in coating porosity (Fig. 2A). Trace amounts of (<1%) Mg were detected in all samples attributed to its presence as a common contaminant of commercially obtained Ca/P materials (Fig. 2A).

An inadequate ability to control Ca:P stoichiometry is a significant shortcoming of the PVD processes such as plasma assisted sputtering. Jansen *et al*. reported Ca deficiencies in coatings sputtered directly from HA targets whilst Boyd *et al*. conveyed an overabundance of Ca^15,31^. Herein, an enhanced understanding of the preferential sputtering phenomena outlined in a previous publication^40^, permitted tailoring of Ca:P ratios by manipulation of working pressures and using a suitable target. Previous publication by Stuart *et al*.^40^ suggested that alkalis such as Ca^2+^, bound into the structure through Ca-O ionic linkages required lower dissociation energies than covalently bound P-O, increasing ejection velocities for alkali components following bombardment of Ar^+^ ions for physical ejection of Ca, P and O. Furthermore a reduction in working argon pressure, was additionally observed to increase alkali:P ratios^40^. Manipulation of these effects allowed customisation of the stoichiometry of Ca/P thin film to produce HA layers that are stoichiometric and crystalline as shown here in step 1: deposition in readiness for step 2: porosification.

In step 2 the post irradiation reduction in Ca/P ratio from 1.67 to 1.43 is likely due to preferred evaporation of Ca in which the decrease of Ca may have been caused by dissociation of the Ca-O and P-O bonds from momentum exchanges during electron irradiation (Fig. 2B) as reported by Matsui *et al*. for electron irradiated metallic surfaces^41^. Comparable dissociation energies of Ca-O and P-O were reported as 464 and 597 kJ mol^−1^ respectively by Dean *et al*. which associates the reduction in Ca to the increase in electron voltage from 13 to 29 kV^42^. In contrast, during melting of CaP phases, boiling points of CaO and P_2_O_5_ of 2850 °C^43^ and 360 °C have led to decrease in P by vaporization^38^.

### Electron Beam Induced Porosification (Surface and Cross Section)

**Figure 3:**
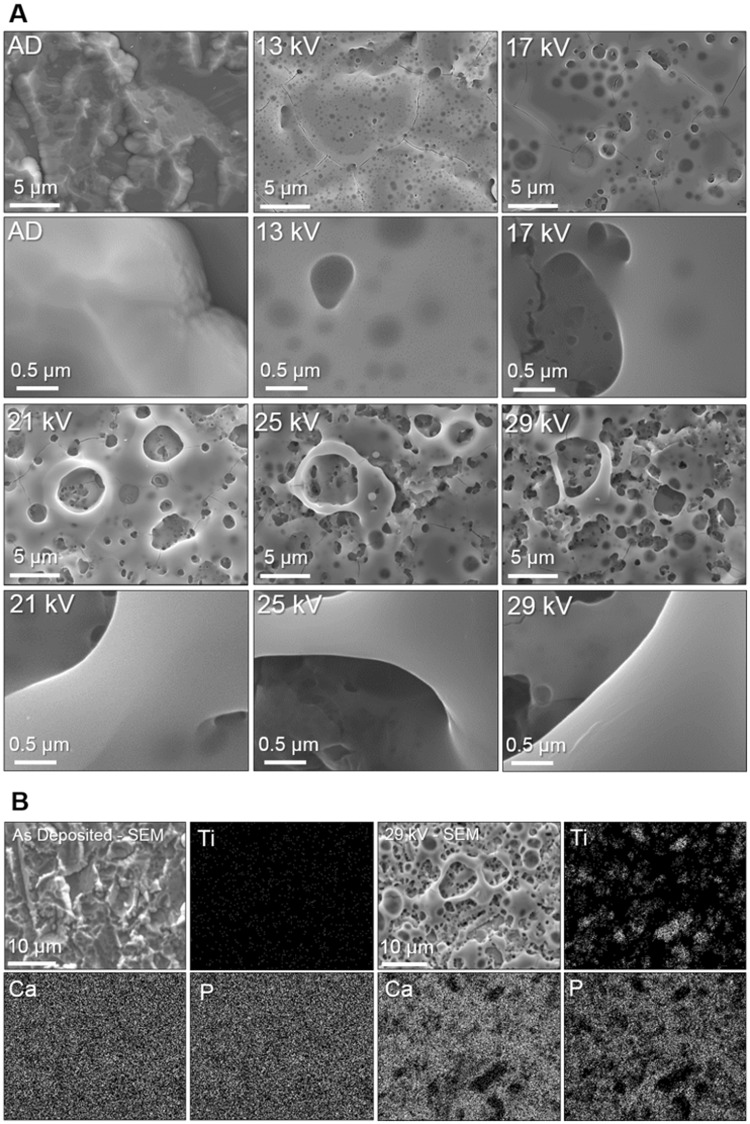
(**A**) SEM of AD and electron irradiated samples displaying an increase in porosity with irradiation voltage. At 13 kV closed porosity was observed within the irradiated layer. High magnification images did not show any noticeably crystal formation within the resolution of the instrument. (**B**) Elemental mapping of AD and 29 kV electron irradiated samples, showing homogenous distributed of Ca/P throughout the coatings pre and post irradiation. 29 kV partially removed complete coating sections as indicated by areas of Ti, corresponding to absence of Ca/P.

Pulsed electron beam irradiation is a unique surface modification process, which uses a large (~60 mm diameter) to cause rapid heating and subsequent cooling of target materials. Typically, the first few microns of metal/ceramic targets become molten, with depth based on penetration of electrons into the material. Cooling rate of the melt is thought to be on the order of 10^7^–10^9^ K s^−1^ ^44,45^, inversely related to energy density. with the effective beam area expected to be uniform within a 60 mm diameter^46,47^. Whilst the determination of internal porosity in thin films is challenging, nano porosity in the 0–10 nm range has been determined in literature via Krypton gas adsorption as shown by Krause *et al*.^48^. Through thickness porosity was indicated here by measuring the change in mass of the film following electron irradiation, indicative of the combination of overall porosity and surface ablation as shown in Fig. 2C. Coating masses were measured post-irradiation, reducing by 0, 1, 3, 24 and 45% for the 13, 17, 21, 25 and 29 kV irradiation respectively, which was attributed to material ablation (Fig. 2C). Scanning electron micrographs (SEM) presented in Fig. 3 showed that the AD coating maintained the morphology of the grit-blasted Ti6Al4V substrate as expected from this technique. LHEB at 13 kV yielded closed internal porosity observed throughout the coating. Subsequent increases in irradiation voltage from 13 kV led to associated increases in porosity, and pore sizes (Fig. 3) with interconnecting and open porosity occurring and increasing above 21 kV. Larger area (1200 µm^2^) SEM micrographs and all 5 randomly selected areas for the 29 kV irradiated sample have been provided as supplementary data to show distribution and morphology of porosity across the sample. Pore area distribution in the Fig. 2D histogram showed sub-micron porosity following 13 kV irradiation, ranging from 0.01–0.69 μm^2^. Maximum pore areas increased from 0.7 to 92.5 μm^2^, subsequently formed by periodically increasing irradiation voltage to 29 kV. However, on all samples the majority of pores led to average pore area distributions ranging from 0.07–1.66 μm^2^ for 13 and 29 kV irradiated samples respectively (Fig. 2E). The linear relationship between average pore area and irradiation voltage suggests potential for the control of pore size by LHEB. In addition, at the higher voltages a few larger pores greater than 10 μm^2^ can be observed as illustrated in Fig. 2D and supplementary images Figs 1 and 2.

Energy dispersive x-ray spectroscopy (EDX) elemental mapping indicated homogenous distribution of Ca/P throughout the AD coating. For comparison, elemental mapping following 29 kV electron irradiation revealed similar homogenous distribution of Ca/P within the porous regions and dispersed across the sample. In some locations the titanium substrate layer was revealed (Fig. 3B).

**Figure 4:**
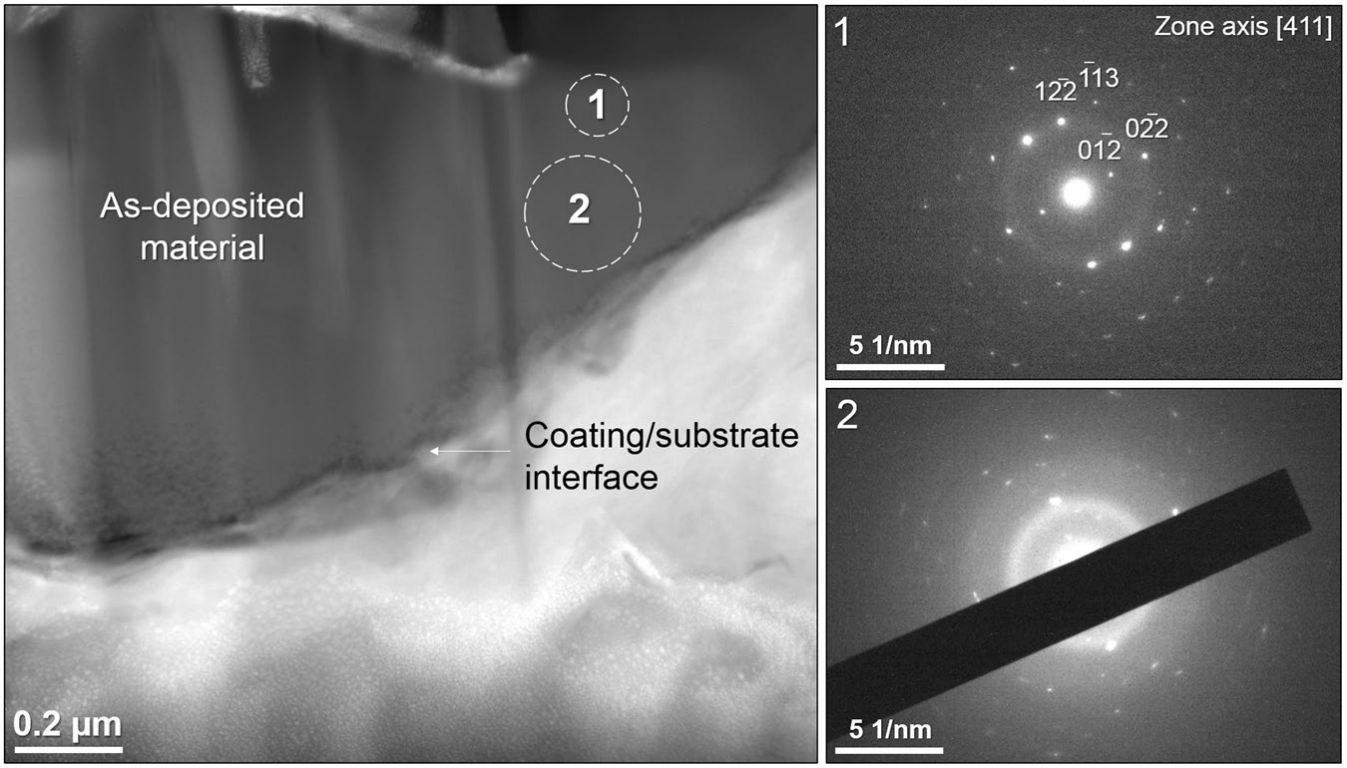
Dark-field STEM image of the unmodified AD coating. SAED patterns were acquired at locations 1 and 2, corresponding to labels with the STEM image.

**Figure 5:**
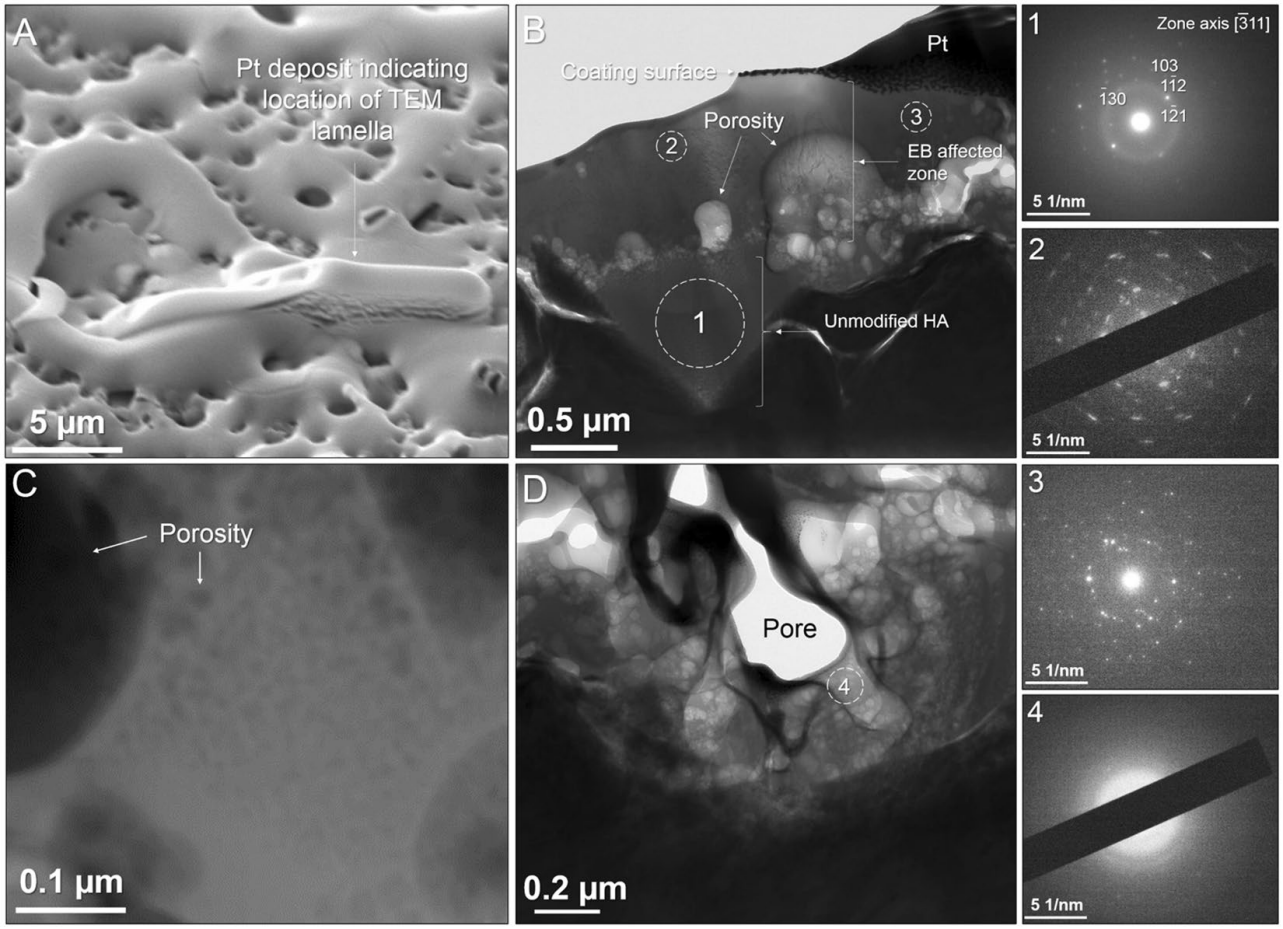
TEM of 25 kV, 1 shot electron irradiated coating. (**A**) Location of FIB lift out lamella extraction (**B**) Dark-field STEM image of high magnification porosity. (**C**,**D**) Bright-field STEM images of complete coating section and magnified porous region. SAED patterns were acquired at locations 1–4, corresponding to labels in C and D.

Cross sectional lamellas were extracted from the AD and 25 kV samples to investigate the effects of LHEB through the coating depth and coating-substrate interface using Transmission Electron Microscopy (TEM) (Figs 4 and 5). The Scanning TEM (STEM) image of the AD coating was absent of any porosity or visible crystal formations whilst conforming to the topography of the substrate (Fig. 4). Selected Area Diffraction Electron (SAED) (Fig. 4 – Location 1), revealed a distinct single orientation crystalline pattern with a faint amorphous structure. Location 2 showed a stronger amorphous signal and multiple crystal orientations contributing to a polycrystalline pattern. Although indication of a residual amorphous phase, commonly reported for sputtered HA films was not observed by XRD, some local evidence was observed by selected area electron diffraction (Fig. 4) using TEM.

Following 25 kV irradiation, a lamella was extracted on the ridge of a larger pore as shown in Fig. 5A. Coating thickness was measured, ranging from 0.8 to 1.7 μm and averaging 1.2 ± 0.1 μm. To help elucidate the range of scales of porosity present within the irradiated coating, a higher magnification dark-field STEM image is shown in Fig. 5B revealing pores with diameter less than 10 nm. The bright-field STEM image (Fig. 5D) of the cross-sectional lamella showed a complex structure of porosity originating from a clear interface *ca*. 1 µm from the top surface of the coating. SAED patterns were acquired *ca*. 2 μm below the surface, beneath the highly porous interface, believed to be unmodified as-deposited material (Fig. 5B - location 1) and matched the diffraction characteristics from the (AD - location 1) sample suggesting an unmodified material beneath the porous interface. Location 1 contained a mixed amorphous/crystalline structure with a single indexable dominant crystal orientation, with some fainter patterns of other crystals, despite the relatively large diameter region of 0.5 µm from which the pattern was taken. SAED was additionally collected from two regions (of ~200 nm diameter) distanced ~500, ~200 nm from the coating surface (Fig. 5B – location 2 and 3 respectively). Both patterns were entirely crystalline with AD indexed to HA along the [3 1 1] zone axis and both samples yielding an array of diffraction spots in a circular pattern, indicative of polycrystalline structures in a random orientation. The existence of multiple orientations from a region of 200 nm diameter is consistent with grain sizes measured via the Scherrer equation – i.e. 62 nm in the case of the 25 kV sample. Figure 5C showed a higher magnification image of a surface-connected large pore near to the coating/substrate interface. This was surrounded by a network of pores, many of which have a diameter of less than 50 nm. A SAED pattern taken from the highly porous region between coating/substrate interface and surface-connected pore in Fig. 5D (Location 4) was indicative of an entirely amorphous structure, given its undefined, broad rings and absence of spots.

Table 2 shows EDX data collected from the TEM, showing a significant drop in Ca/P ratio in the modified zone of the coating after irradiation. The unmodified region (Fig. 5 location 1) reveals only a minor change in Ca/P ratio, reflecting the unmodified microstructure in this location. It should be noted that the TEM lift out process may cause small alterations to chemical compositions due to gallium ion milling and the more significantly in this case, due to the direct overlap of the Platinum Mα X-Ray emission with Phosphorus Kα emission at 2.05 and 2.01 KeV respectively during EDX analysis. The platinum band is deposited during the lift out methodology^49,50^.

**Table 2:** Atomic % EDX data from cross-sectional TEM as-deposited and 25 kV irradiated samples, confirming drop in Ca/P ratio in modified region of coating. Locations numbers correspond to Figs 4 and 5.

	Atomic %			
	Ca	P	O	Ti
As-deposited location 2	36.7	24.7	37.4	1.2
25 kV location 1	34.8	24.7	39.8	0.64
25 kV location 2	28.0	20.1	52.0	0.0

SAED patterns 2 and 3 shown in Fig. 5 confirm that a polycrystalline structure, with no amorphous signal dominated the irradiated coating in regions without pores, even closest to the top surface. From prior work it has been shown that the finest grains, implying fastest cooling rates are produced next to the top surface^51^. However, for example in pattern 4 an entirely amorphous structure was present in a region dominated by pores. Therefore, it is thought that only cooling rates yielded by material which undergoes melt expansion are high enough to cause amorphous phase formation. For all other regions, the cooling rate was low enough to retain a fine, randomly oriented polycrystalline structure.

Cross-sectional TEM imaging showed that porosity in the irradiated sample originated from an interface (*ca*. 1 μm from the top surface) between the electron beam modified coating region and the underlying unmodified material (Fig. 5B). Pore expansion appears to have radiated towards the top surface. Individual crater formation in materials subject to LHEB irradiation has been well characterised from the surface and it has been proposed that the dominant mechanism is the presence of irregularities of chemical or phase composition leading to localised, sub-surface melting below the melting threshold of the matrix. This is followed by expansion and breakthrough of the molten material through a more rapidly solidified top surface layer. In materials tested so far, crater formation tends to be sparse and with a uniform morphology. In the case of the Ca/P material in this work, however, porosity is non-uniform, complex and interconnected. The amorphous nature of material closer to the substrate is attributed to faster cooling as the Ti6Al4V substrate acted as a heat sink. The TEM image in Fig. 5B revealed pores present within the cross-section with a large range of sizes. A large pore could be seen with diameter of *ca*. 600 nm, surrounded by many smaller pores. This can be contrasted with other nearby regions in which finer pores were present, as well as regions where a relatively large pore is present surrounding a network of smaller pores (Fig. 5B). In addition, Fig. 5D showed that the largest pore present within the TEM lamella, which is void of any material, was surrounded by a network of fine pores. Therefore this gave evidence that the most severe porosity originated from the nucleation of extremely fine porosity well below 50 nm in diameter. The agglomeration of the expanding material pores driven by surface energy reduction results in much larger pores which can penetrate the top surface. It is suggested that pore nucleation originated at maximum electron beam penetration at a depth of *ca*. 1 μm (25 kV). This increased penetration depth correlates with electron acceleration voltage and would therefore yield larger pore sizes relative to the cathode voltage parameter.

**Figure 6:**
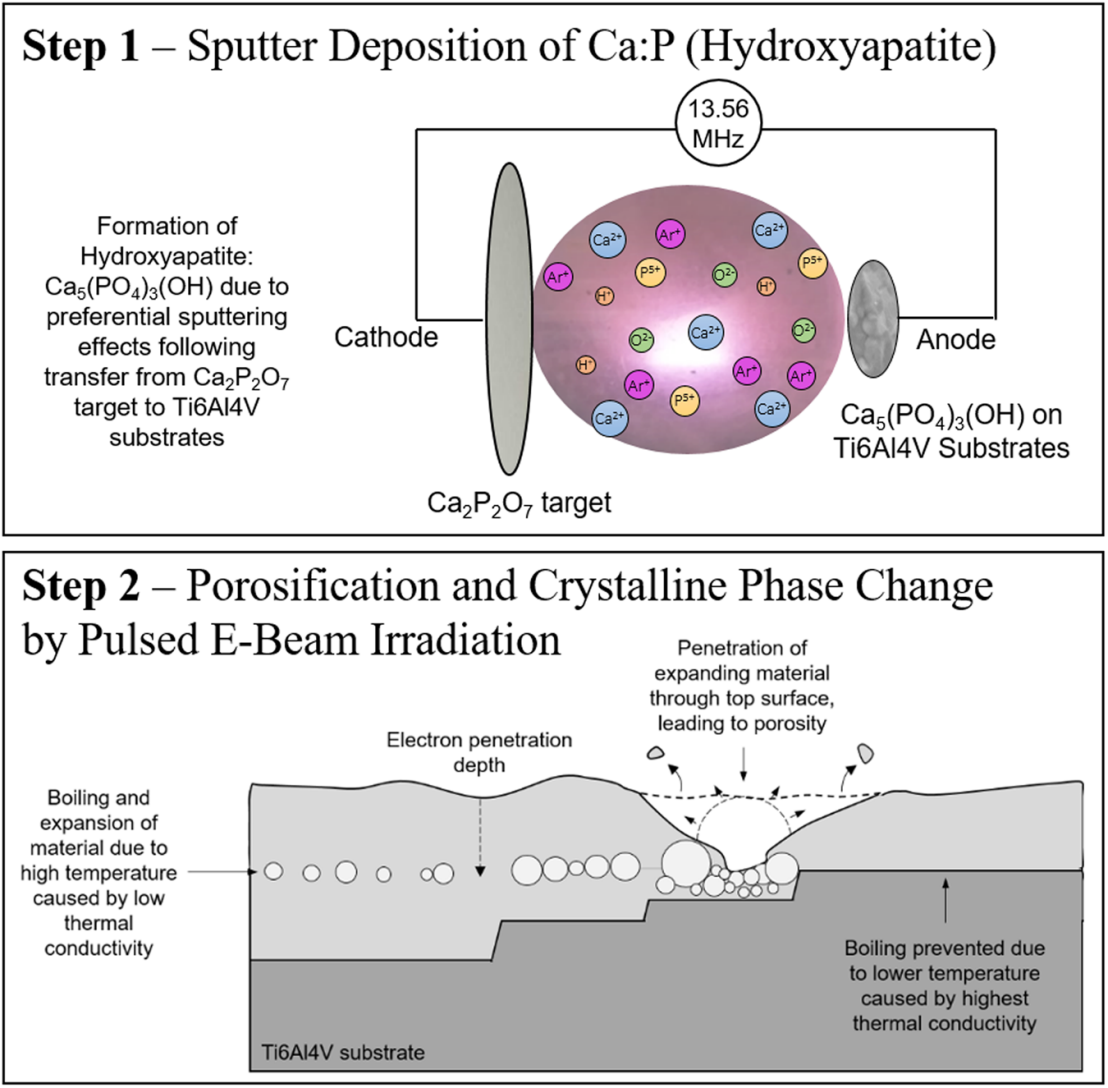
Schematic representation of step 1: deposition of HA onto Ti6Al4V substrates from preferential sputtering effects following transfer from the Ca_2_P_2_O_7_ target and step 2: mechanisms for coating porosification following electron beam irradiation. The electron beam penetrates to the interface of HA and Ti6Al4V. Boiling of the coating leads to localised expansion and porosification. The Ti6Al4V substrate acted as a heat sink, thereby causing amorphous substrate/coating interfacial regions whilst slower cooling near the surface formed a polycrystalline structure. When the interface layer is just less than the coating depth then the agglomerated pores burst through the surface and expose the Ti alloy substrate.

The graphical schematic presented in (Fig. 6) shows the two step process by sputtering and electron beam irradiation respectively as well as the proposed mechanism of porosification caused by LHEB and heat dissipation via the Ti6Al4V substrate heat sink due to differential thermal conductivities between HA and Ti6Al4V (of 0.7 W/m.K and 8–17 W/m.K respectively)^52,53^.

Temperature within the coating is highest at maximum penetration depth of the electrons. Beneath this point, the unaffected HA acts as a poor heat sink owing to its low thermal conductivity. This build-up of molten material expands in the direction of the surface through the molten but cooling material. The top surface is expected to cool first and hence be solidified when porosity expansion reaches the top surface, resulting in explosive crater formation. The complex range of pores can be explained by differences in thickness of material beneath the penetration depth of electrons. TEM imaging revealed that the most intensive sub-surface porosity, which leads to the most severe surface porosity, is in locations in which the remaining HA coating beneath the molten regions is thinnest, thus the coating is just thicker than the boiling depth. In this scenario the agglomerating pores, when sufficiently large can burst through the surface. In the case that the penetration depth of electrons reaches the underlying Ti6Al4V substrate, i.e. the coating is thinner than the optimum boiling depth then boiling does not take place owing to the much increased heat dissipation through the order of magnitude higher heat conducting Ti6Al4V alloy.

## Conclusions

Here we have developed a new two step process to generate controlled porosity of Ca/P coatings that provide a pseudo 3-D mimic of bone. The first step uniquely generated a crystalline HA surface using plasma assisted PVD sputtering from a crystalline Ca:P oxide target. The second step uses a new facile and rapid post treatment by a single shot pulsed large area electron irradiation technique. Utilising LHEB with voltages from 13–29 kV led to the new discovery of a method to generate a complex interconnected porous layer based on osteoconductive α-TCP with a morphology similar to that of bone. Extent of porosity was shown to be controllable via parameter control, with a maximum mass loss of 45% and increase in average/maximum pore sizes from 0.07–1.66/0.69–92.53 μm^2^. Simultaneous dissociation of Ca led to a reduction in Ca:P ratio from 1.67 to 1.43, synonymous with HA and calcium deficient TCP phases, as well as incremental conversion of HA to α-TCP, irradiation voltage dependent, revealed via XRD and TEM, suggesting flash melting of the structure to above 1125 °C based on phase transformation of Ca:P mixtures. A randomly oriented polycrystalline structure was seen in non-porous regions of the 25 kV irradiated coating, with no amorphous phase, with porous regions being highly amorphous suggesting rapid cooling. Severe porosity originated from the interface between maximum electron penetration depth and unaffected material below, with expansion of pores towards the surface and breaking through to yield significant surface porosity. Correlation of porosity with electron acceleration voltage can be explained by the increased penetration depth, thereby giving more lateral expansion of material prior to surface break-through. This new two step methodology can be readily applied to most metallic surfaces, with the additional advantage that selected areas can be single shot pulsed by LHEB allowing regions to be treated and untreated. This is ideal for hip stems where full integration is not required otherwise implant recovery causes too much damage. Insensitivity of the LHEB process to large incident angles also makes the process suited to complex shapes. Thus the manufactured porosity and ability to manipulate crystallinity of Ca:P thin films by a combination of PVD and LHEB shows great potential to produce surface scaffold-like coatings on orthopaedic implants as a suitable layer for bone integration and may have further uses in thin-film electronics or ceramic membrane applications.

## Experimental Methods

### Physical Vapour Deposition - Magnetron Sputtering

A Ca_2_P_2_O_7_ target, with an intended Ca/P ratio of 1.0 was obtained by mixing 90.6 g of (98–105% Purity) Calcium Phosphate Dibasic (CaHPO_4_) (sigma Aldrich) with 0.1 g of (>98% Pure) Phosphorous Pentoxide (P_2_O_5_) (fisher scientific). The mixture was dehydrated at 400 °C for 30 min in a Pt:Rh 90:10% crucible followed by melting for 2 h at 1370 °C and quenching to room temperature. The quenched product was milled to a powder for ~20 min at 400 RPM using 3 alumina balls and mechanically sieved to <64 μm. The powders were subsequently pressed into an aluminium powder cup to produce the Ca_2_P_2_O_7_ target of 75 mm diameter and 4 mm in thickness.

Coatings were deposited on Ti6Al4V substrates via a custom in-house designed magnetron sputtering rig built at the University of Nottingham. Ti6Al4V substrates were alumina grit blasted, as previously set out in^25^. The chamber was pumped down by combination of a rotary (Edwards E2M-18) and turbo-molecular (Edwards EXT250) pumps to a base pressure below 7 × 10^−3^ Pa. The substrates were fixed at a distance of 40 ± 2 mm from the target surface. The argon pressure (purity 99.99%, BOC^©^) was maintained at 2.5 mTorr/20 SCCM via a mass flow controller (MKS 2982) and an (MKS 250) pressure controller and monitored by a temperature controlled capacitive pressure transducer (MKS Baratron 627B). The Ca_2_P_2_O_7_ target was deposited by ion bombardment using a Radio Frequency (RF) (13.56 MHz) power supply with an 80 W deposition power.

### Low-energy High-current Pulsed Electron Beam Irradiation (LHEB)

A Sodick PF32A EBM machine was used for electron beam irradiation experiments. The irradiation process was carried out in an air-tight chamber into which an argon at a pressure of 0.05 Pa was pumped. Argon gas is used as the medium for plasma build up required for the electron generation and beam propagation. Bombardment of the high current electrons with a workpiece causes rapid heating and cooling at the near surface of the workpiece material. A range of cathode voltages and single pulses was used for irradiation tests. Repeat samples were irradiated in the same batch to ensure consistent beam properties. Cathode voltage determines acceleration and hence energy density of the electrons. Parameters are shown in Table 3.

**Table 3:** Pulsed electron beam irradiation parameters.

Cathode voltage (kV)	Energy density (J/cm^2^)	Number of pulses	Anode voltage (kV)	Solenoid voltage (kV)	Argon pressure (Pa)	Irradiation distance (mm)
13, 17, 21, 25, 29	~0.2, 0.9, 2.1, 3, 10.5	1	5	1.5	0.05	300

### Mass Loss – Porosity Measurement

Samples of alumina blasted Ti6Al4V were weighed pre coating, post coating and post irradiation using a Sartorius ME36S microbalance, accurate to 1 μg to determine the fractional mass loss due to electron irradiation. Porosity was calculated via an automated thresholding method in ImageJ software for pores present in the case of the 13 kV sample, and was measured via manual tracing of each pore in the case of all other samples. Pore areas below 0.01 µm were not measured in all cases due to poor SEM resolution at this scale. Porosity measurements were taken from the average of 5 randomly selected, 1200 μm^2^ SEM images from a 78 mm^2^ sample area (10 mm diameter substrate size).

### Scanning Electron Microscopy (SEM) – Energy Dispersive X-Ray Spectroscopy (EDX)

Electron micrographs were collected using a JOEL 7100 F Field Emission Gun - Scanning Electron Microscope (FEG-SEM). All images were acquired with a beam voltage of 15 kV at a working distance of 10 mm. EDX analysis was performed with an attached Oxford Instruments X-Ray detector by scanning for a minimum of 500,000 Counts over a 1600 μm^2^ region.

### X Ray Diffraction (XRD)

Grazing Incidence XRD patterns were acquired on a Bruker D8, (Cu Kα source, λ = 1.5418 A, 40 kV, 40 mA) over the 2θ range from 20° to 40°. The X-Ray incident angle of 2° was set to avoid excessive substrate signal. Scans were acquired using a step size of 0.01° in 2θ and dwell time of 25 s. Crystallite sizes were calculated using the Scherrer equation by converting all angles to radians and using an instrumental broadening value of 0.129. The FWHM was calculated by fitting Gaussian distributions using OriginPro 2018.

### Focused Ion Beam and Transmission Electron Microscopy

Focused Ion Beam (FIB) machining using an FEI Quanta 200 3D FIBSEM was performed to prepare lamellas for Transmission Electron Microscopy (TEM). Protective platinum deposits were used in the location of the extracted lamellas. TEM and Scanning TEM (STEM) were performed with a JEOL 2100 F at 200 kV. Bright field (BF) and Dark Field (DF), STEM were performed using a JEOL Digital STEM System. Energy dispersive X-ray spectroscopy (EDX) mapping was performed in STEM mode using an Oxford Instruments INCA X-Ray Microanalysis System. Hydroxyapatite and Tri Calcium Phosphate were indexed using CrystBox software and crystallographic data from card nos. 2300273 and 9005865 respectively, from the Crystallography Open Database (http://www.crystallography.net).

## Electronic supplementary material

Supplementary Information
