# Supplementary material for: Two step porosification of biomimetic thin-film hydroxyapatite/alpha-tri calcium phosphate coatings by pulsed electron beam irradiation
Source: Sci Rep. 2018 Sep 28;8:14530. doi: 10.1038/s41598-018-32612-x (PMC6162225; doi:10.1038/s41598-018-32612-x)
Supplement: Supplementary file 1 — Supplementary Information [file 41598_2018_32612_MOESM1_ESM.docx]

**Two step porosification of biomimetic thin-film hydroxyapatite/alpha-tri calcium phosphate coatings by pulsed electron beam irradiation**

**Bryan W. Stuart*^1^, James W. Murray^1^, David M. Grant^1^**

^1^ Department of Mechanical, Materials and Manufacturing Engineering (Advanced Materials Research Group), University of Nottingham, UK

**Supplementary Images –**


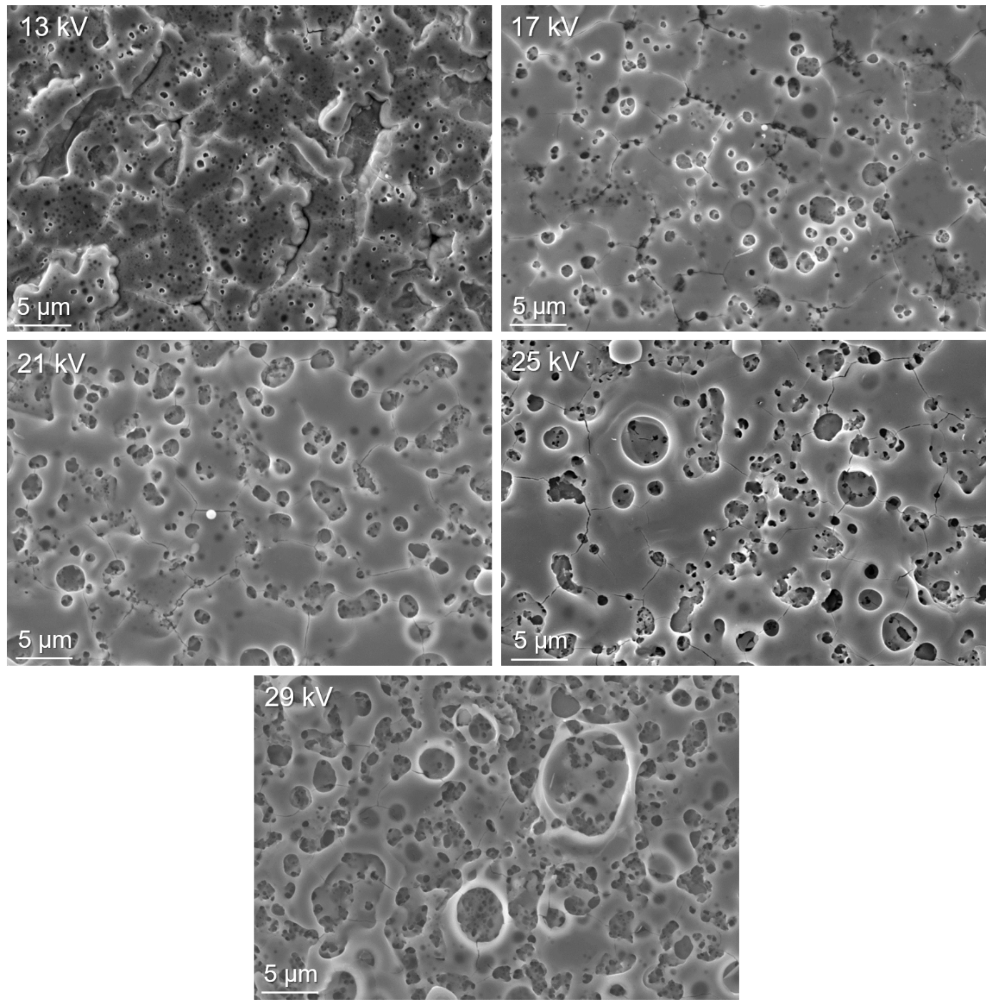


**Supplementary Figure 1 Example SEM images of coatings irradiated at different voltages, used to calculate porosity data**


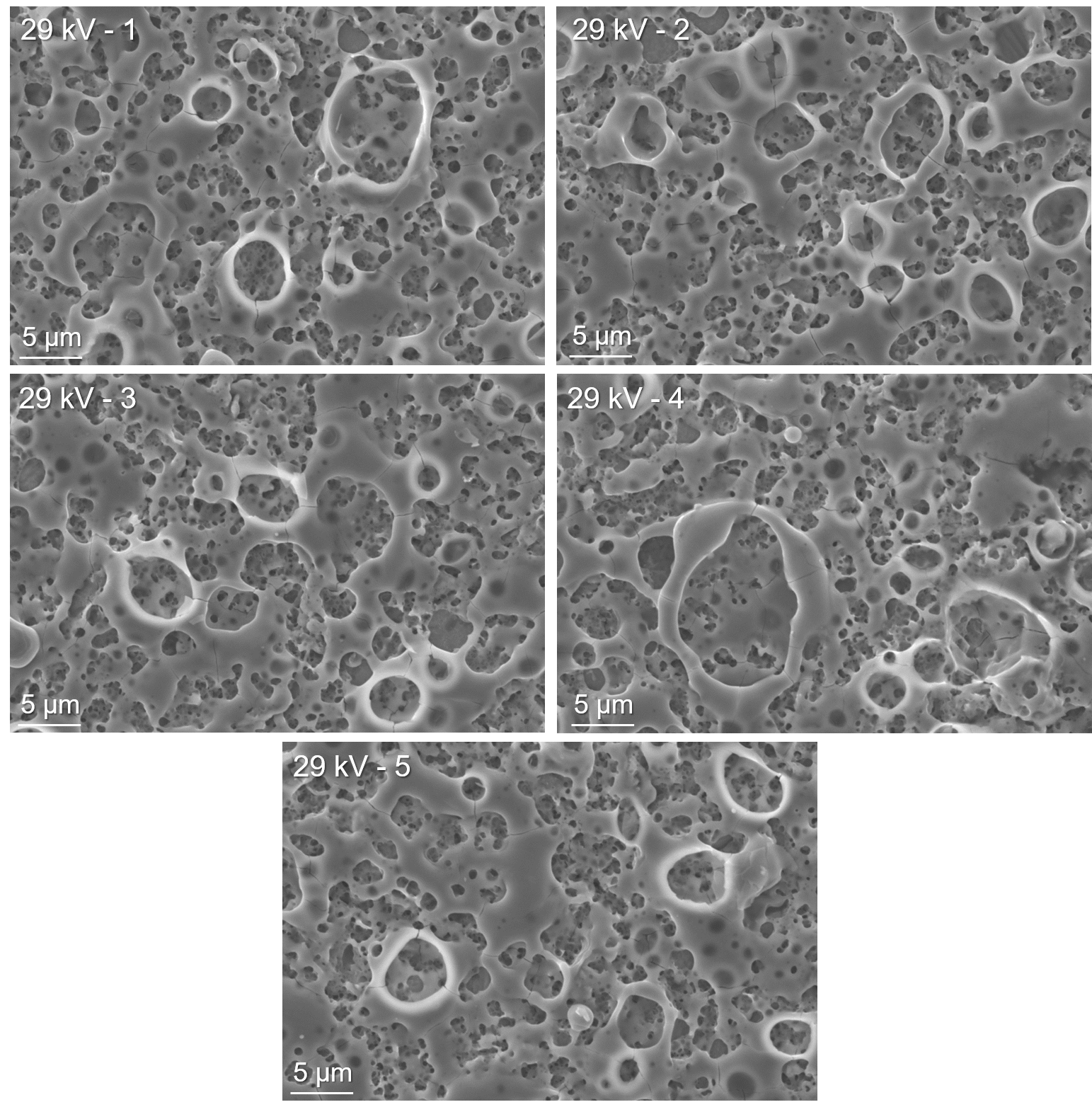


**Supplementary Figure 2 all five SEM images of the 29 kV irradiated coating at different sample locations, showing consistency of morphology across the sample.**
